# Homœopathy and Gynæcology

**Published:** 1887-01

**Authors:** 


### BOOK NOTICES.

HOMŒOPATHY AND GYNÆCOLOGY. By Thomas Skinner, M. D. The Homœopathic Publishing Company. London. 1886.

Dr. Skinner's interesting brochure upon the relations of Homœopathy to the diseases of females is too well known to need a word from us. That he has found it necessary to issue a third edition attests its popularity. This brochure is one of the best our school possesses to place in the hands of female patients, who doubt the efficacy of Homœopathy in treating their complaints. Woman have been so miserably misled (mistaught) by the so-called gynæcologists of the day, that they believe they must needs be treated locally to be cured.

Dr. Skinner declares (and many other Hahnemannians will indorse his declaration) that: "Constitutional treatment alone is all that is necessary for the successful treatment of all vaginal, uterine, ovarian, and pelvic diseases," which statement he supports by narration of a few cases.

